# Supplementary material for: Prediction of Overall Survival by Thymidine Kinase 1 Combined with Prostate-Specific Antigen in Men with Prostate Cancer
Source: Int J Mol Sci. 2023 Mar 8;24(6):5160. doi: 10.3390/ijms24065160 (PMC10049218; doi:10.3390/ijms24065160)
Supplement: Supplementary file 1 [file ijms-24-05160-s001.zip › ijms-2210181-supplementary.pdf]

Supplementary Materials

Table S **1**

		Age at	Age at			ng/ml		ng/ml/cm^3	ng/ml/cm^3	ng/ml		
**Time of diagnosis, years**		**Ageat screening**	**Ageat diagnosis**	**Ageat last follow-up**	**Alive,** %.	**PSA, nglml**	**Free/total,** cm^3·	**Volume, nglml/cm'·**	**PSA density, nglml/ cm'**	**TKI*, nglml**		**Other malign, %**
±1	n=65	64.5 (5.8)	64.9 (5.3)	77.2 (10.7)	7.7	7.5 (16.8)	0.08 (0.07)	25.6 (12.6)	0.29 (0.54)	0.26 (0.17)	52	15.4
1-5	n=l5	66.8 (3.6)	69.4 (3.3)	79.2 (15.8)	6.7	3.8 (8.0)	0.15 (0.12).	19.3 (10.0)	0.13 (0.30)	0.29 (0.14)	11	13.3
5-10	n=37	64.4 (5.9)	72.4 (6.0)	80.4 (11.8)	16.2	4.6 (4.7)	0.14 (0.09)	23.7 (11.0)	0.15 (0.12)	0.25(0.16)	28	16.2
lG-15	n=56	63.5 (6.9)	75.9 (7.0)	84.4 (7.3)	21.4	3.3(3.6)	0.15 (0.08)	25.2 (13.1)	0.13 (0.10)	0.25(0.12)	31	21.4
15-20	n=66	61.2 (6.7)	78.9 (6.4)	86.6 (5.4)	33.3	2.1 (2)	0.16 (0.10)	21.4(9.6)	0.11 (0.09)	0.22 (0.12)	34	21.2
2G-30	n=32	59.4 (5.5)	83.9 (7.0)	88.1 (4.2)	43.8	1.9 (1.4)	0.15 (0.08)	22.0 (11.0)	0.09 (0.05)	0.26 (0.16)	19	12.5

*n=l75

Table S 1. Age composition and baseline data in 271 men with prostate cancer in relation to time of diagnosis during 30 years of follow-up. Median values (IQR). Abbreviations: PSA = prostate specific antigen, TKl = thymidine kinase 1

Table S 2

	**With TKl** (n = 175)	**WithoutTKl** (n = 96)	p
Age	63.4 (5.9)	62.9 (8.0)	0.169
PSA, ng/ml	3.8 (5.4)	2.6 (3.4)	0.017
Free/total PSA.	0.13 (0.10)	0.12 (0.09)	0.110
Prostate volume, cm^3·	23.0 (12.0)	24.2 (12.2)	0.778
PSA density, ng/ml/cm^3	0.14 (0.19)	0.13 (0.10)	0.048
Alive, %	19.4	22.9	0.500
Other malignancies, %.	18.3	16.7	0.738

Table S 2. Baseline data of 275 men with prostate cancer. TKl concentration was measured in 175 men and was missing **in** 96 men. Mean values (IQR).

Figure S 1.

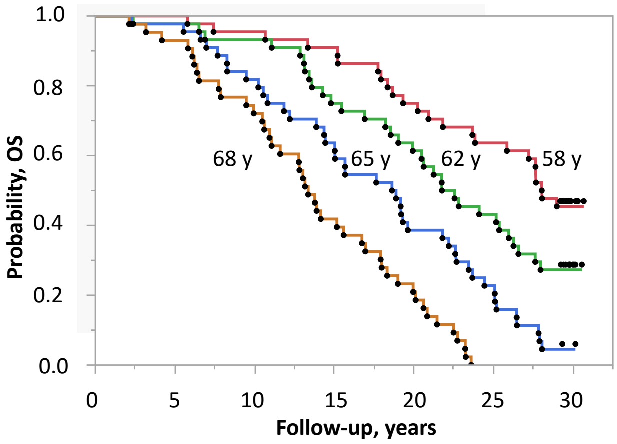

Figure S 1. Overall survival of 175 men with prostate cancer stratified by quartiles of age at screening. Kaplan-Meier estimates, median survival (95% CI) 28.0 (23.7-), 22.6 (19.0-26.3), 18.1 (13.9-21.8) and 13.6 (10.9-17.0) years.

Figure S 2.

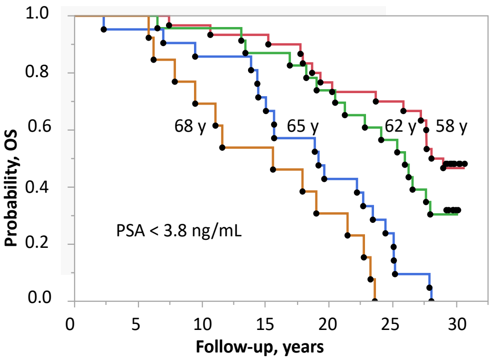

(a)

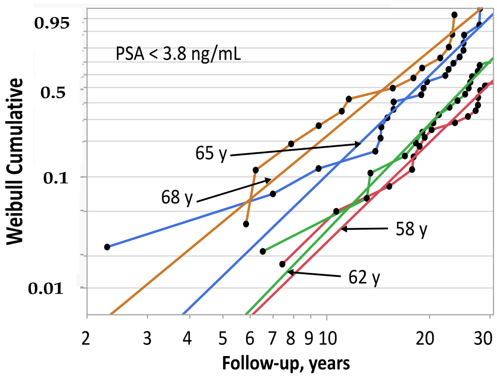

(b)

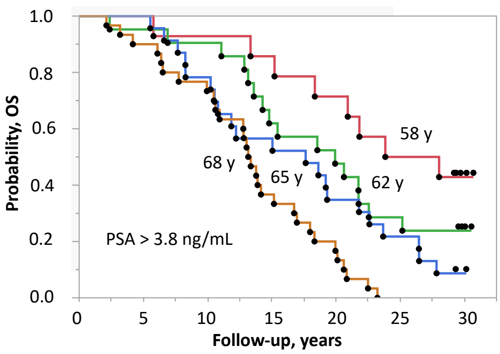

(c)

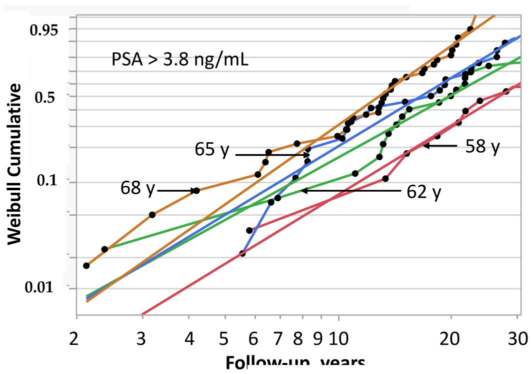

(d)

Figure S 2. Overall survival of 175 men with prostate cancer stratified by quartiles of age at screening and median PSA concentration: (a) Kaplan-Meier estimates of PSA concentrations <3.8 ng/ml; (b) Weibull transformed Kaplan-Meier estimates of PSA concentrations <3.8 ng/ml; (c) Kaplan-Meier estimates of PSA concentrations >3.8 ng/ml; (d)Weibull transformed Kaplan-Meier estimates of PSA concentrations >3.8 ng/ml

Table S 3.

**Age** (mean, 95%CI)	**TK1 < 0.25, ng/ml** (95% CI)	**TK1 >0.25, ng/ml** (95% CI)	**Ratio** < 0.25/>0.25	**PSA <3.8** (95% CI)	**PSA >3.8** (95% CI)	**Ratio** <3.8/>3.8
58.1 (57.6-58.5)	32.5 (28.9-41.0)	30.8 (24.8-42.6)	1.06	32.7 (28.2-41.5)	31.8 (23.7-53.8)	1.03
61.9 (61.7-62.2)	29.6 (25.5-36.7)	22.3 (17.9-28.6)	1.33	28.2 (24.4-34.1)	23.9 (18.6-32.2)	1.18
65.0 (64.7-65.3)	23.0 (20.1-26.5)	17.4 (14.9-21.3)	1.32	20.6 (17.6-24.0)	19.7 (15.9-24.5)	1.05
68.3 (68.0-68.7)	15.6 (12.8-18.8)	15.5 (12.9-18.5)	1.01	17.0 (13.2-21.5)	14.9 (12.7-17.4)	1.14

Table S 3. Weibull survival probability of TK1 and PSA below and above median concentration and ratio of TK1 <0.25/>0.25 ng/ml and PSA <3.8/>3.8 ng/ml in 175 men with prostate cancer stratified by 4 age groups.

Figure S 3.

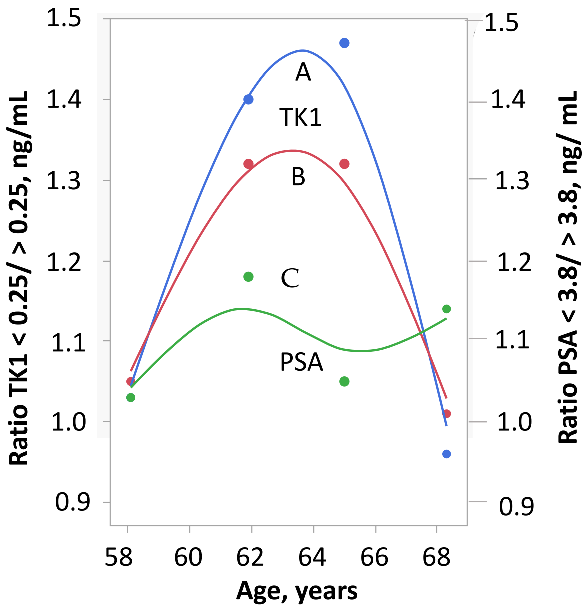

Figure S 3. Ratio of TK1 <0.25/>0.25 ng/ml and PSA <3.8/>3.8 ng/ml in 4 age groups of 175 men with prostate cancer. **A**: Median survival of Kaplan-Meier estimates of TK1, **B**: Weibull survival probability of TK1, **C**: Weibull survival probability of PSA.

Figure S 4.

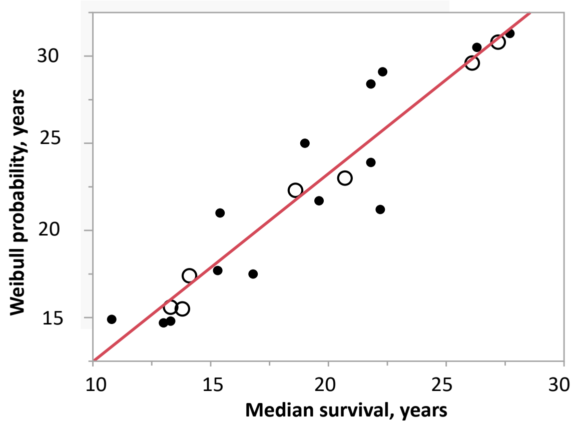

Figure S 4. Relationship between median survival time and survival probability calculated from Weibull transformed Kaplan-Meier survival estimates of 175 men with prostate cancer and 30 years of follow-up. Open symbols: subgroups of 14 observations, closed symbols: subgroup of 7 observations.
